# Antibacterial Activity of the Flavonoids from *Dalbergia odorifera* on *Ralstonia solanacearum*

**DOI:** 10.3390/molecules16129775

**Published:** 2011-11-25

**Authors:** Xiabo Zhao, Wenli Mei, Mingfu Gong, Wenjian Zuo, Hongjin Bai, Haofu Dai

**Affiliations:** 1 Hainan Key Laboratory for Research and Development of Natural Products from Li Folk Medicine, Institute of Tropical Bioscience and Biotechnology, Chinese Academy of Tropical Agricultural Sciences, Haikou, Hainan 571101, China; 2 Xinjiang Production and Construction Corps Key Laboratory of Protection and Utilization of Biological Resources in Tarim Basin, College of Life Science, Tarim University, Alar, Xinjiang 843300, China; 3 College of Chemistry and Life Science, Leshan Normal University, Leshan, Sichuan 614000, China

**Keywords:** *Dalbergia odorifera*, flavonoids, antibacterial activity, anti*-Ralstonia solanacearum*

## Abstract

Phytohemical investigation on the heartwood of *Dalbergia odorifera* resulted in the isolation of nine flavonoids. Their structures were elucidated as sativanone (**1**), (3*R*)-vestitone (**2**), (3*R*)-2',3',7-trihydroxy-4'-methoxyisoflavanone (**3**), (3*R*)-4'-methoxy-2',3,7-trihydroxyisoflavanone (**4**), carthamidin (**5**), liquiritigenin (**6**), isoliquiritigenin (**7**), (3*R*)-vestitol (**8**), and sulfuretin (**9**) based on their spectral data. All compounds were evaluated for their inhibitory activity against *Ralstonia solanacearum*. This is the first report about anti-*R. solanacearum* activity of the compounds from *D. odorifera*.

## 1. Introduction

*Ralstonia solanacearum*, the pathogen that is the causal agent of bacterial wilt, is one of the best-known bacterial diseases, and is found in tropical, subtropical, and some temperate regions of the World. This soilborne pathogen attacks more than 200 plant species, including many agriculturally important crops [1]. This bacterium can also be free-living as a saprophyte in water or in the soil in the absence of host plants [2]. Streptomycin is widely used in agriculture, but the overuse of it can lead to bacterial resistance [3]. Thus, it is very necessary to search for more potent anti-*R. solanacearum* compounds.

The heartwood of *Dalbergia odorifera* T. Chen, named “Jiangxiang” in Chinese traditional medicine, was used in China and Korea for the treatment of blood stagnation syndrome, ischemia, swelling, necrosis and rheumatic pain [4,5]. Previous chemical investigations on this plant have led to the isolation of flavonoids and phenolic compounds [6,7,8]. Some flavonoids have been reported to possess various pharmacological effects such as anti-inflammatory, antibacterial, antiplasmodial, antinephritic, neuroprotective and antioxidant activities [9,10,11,12,13,14]. During the course of our screening for anti-*R. solanacearum* agents from tropical medicinal plants, the crude ethanol extract of the heartwood of *D.odorifera* showed anti-*R. solanacearum* activity. In this paper, we described the isolation, identification and anti*-R. solanacearum* activity of compounds **1**−**9**.

## 2. Results and Discussion

**Figure 1:**
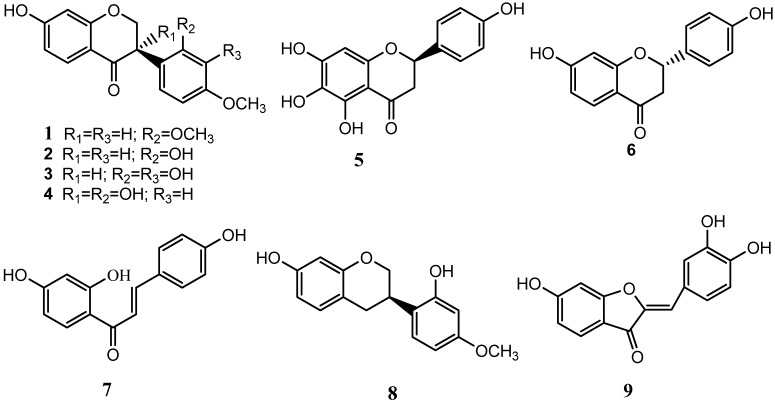
Structures of compounds **1**−**9**.

The compounds (Figure 1) were identified as: sativanone (**1**), (3*R*)-vestitone (**2**), (3*R*)-2',3',7-trihydroxy-4'-methoxyisoflavanone (**3**), (3*R*)-4'-methoxy-2',3,7-trihydroxyisoflavanone (**4**), carthamidin (**5**), liquiritigenin (**6**), isoliquiritigenin (**7**), (3*R*)-vestitol (**8**), and sulfuretin (**9**) by comparison of their spectral data with the literature.

Compounds **1**−**9** were next evaluated for their inhibitory activity against *R. solanacearum *(Table 1). Among the nine flavonoids, compound **8** exhibited the strongest antibacterial activity, with an inhibition zone diameter of 16.62 mm, which was close to that of streptomycin sulfate (the positive control). Compounds **2**, **6** and **7** also showed stronger antibacterial activities than the rest of compounds, with inhibition zone diameters of 11.19, 12.23, and 14.15 mm, respectively.

**Table 1:** Antibacterial activity of compounds **1**−**9**from *Dalbergia odorifera* (mm).

Compound	*Ralstonia solanacearum*	Compound	*Ralstonia solanacearum*
1	6.53 ± 0.05	6	12.23 ± 0.45
2	11.19 ± 0.15	7	14.15 ± 0.95
3	8.11 ± 0.14	8	16.62 ± 1.07
4	9.99 ± 1.25	9	9.10 ± 1.22
5	8.34 ± 0.16	Streptomycin sulfate ^a^	16.80 ± 0.33

The results of diffusion method are presented as diameters of inhibition zones in mm. Each value represents mean ± SD (n = 3). ^a^ Streptomycin sulfate was used as positive control.

Compounds **1**−**4** belong to the isoflavanone class. Compound **1** showed lower activity than the other compounds, and this may be due to the absence of the 2'-OH group, suggesting that this 2'-OH is a favorable group for activity. Compounds **2**−**4** had a B-ring OH group (2' position), and **3** had a B-ring OH group (3' position), while **4** had a C-ring OH group (**3** position). Lower activity of **3**compared to that of **2** seemed to be because the 3'-OH and 2'-OH formed a stable five-membered ring, which reduced the inhibition of the 2'-OH group. Compound **4** had slightly reduced inhibition compared with **2**, which leads us to speculate that the 3-OH and 2'-OH formed an unstable six-membered ring. Compound **8** belong to the isoflavane class which lack the C(4)=O in the C-ring compared with **2**, and its activity was higher than that of **2**. The result suggests that the presence of C(4)=O will reduced the inhibitory effect. 

## 3. Experimental

### 3.1. General

The NMR spectra were recorded on a Bruker AV-400 spectrometer, using TMS as an internal standard. Column chromatography was performed with silica gel (Marine Chemical Industry Factory, Qingdao, China) and Sephadex LH-20 (Merck). TLC was preformed with silica gel GF254 (Marine Chemical Industry Factory, Qingdao, China) plates.

### 3.2. Plant Materials

The dried heartwood of *D. odorifera* was purchased from the Haikou Free Market of Agricultural Products, Hainan Province, China, in October, 2010. The specimen was identified by Professor Zheng-fu Dai of the Institute of Tropical Bioscience and Biotechnology, Chinese Academy of Tropical Agricultural Sciences, where a voucher specimen (No. 20101009) has been deposited.

### 3.3. Extraction, Fractionation and Identification of the Flavonoids

The dried and crushed heartwood of *D. odorifera* (8.4 kg) was extracted three times with 95% ethanol (50 L) at room temperature for three weeks totally. The ethanol extract was then filtered through absorbent gauze, and the filtrate was concentrated on a rotary evaporator under reduced pressure at 50 °C to remove ethanol, resulting in a crude ethanolic extract. This was partitioned with petroleum ether, ethyl acetate and *n*-butanol. The ethyl acetate phase (477.0 g) was submitted to column chromatography (CC) over silica gel eluted with a mixture of chloroform and methanol (100:1–0:100, v/v) of increasing polarity resulting in eighteen fractions (Fr.1–Fr.18). Compound **1** (100.0 mg) obtained by recrystallization from Fr.6 (52.0 g). Repeated CC on silica gel CC eluted with CHCl_3_-MeOH (100:1–0:1, v/v) and Sephadex LH-20 (CHCl_3_-MeOH, 1:1, v/v), led to the isolation of compounds **2** (34.0 mg), **3** (4.0 mg), **4** (64.4 mg), **5** (5.0 mg), **6** (10.4 mg), **7** (70.0 mg) and **8** (5.0 mg) from Fr.10 (45.0 g). Fr.12 (51.3 g) was submitted to column chromatography over silica gel eluted with CHCl_3_-MeOH (50:1–0:1, v/v) and further purification with Sephadex LH-20 (95% EtOH) to afford compound **9** (7.6 mg). The physicochemical and spectrometric data of nine flavonoids were as follows: 

*Sativanone* (**1**). White powder; C_17_H_16_O_5_; ^1^H-NMR (CD_3_OD), *δ*: 4.40 (1H, dd, *J* = 11.0, 5.5 Hz, H-2a), 4.54 (1H, d, *J* = 11.0 Hz, H-2b), 4.16 (1H, dd, *J* = 11.0, 5.5 Hz, H-3), 7.76 (1H, d, *J* = 8.7 Hz, H-5), 6.49 (1H, d, *J* = 8.7 Hz, H-6), 6.51 (1H, s, H-8), 6.33 (1H, d, *J* = 2.3 Hz, H-3′), 6.46 (1H, dd, *J* = 8.4, 2.3 Hz, H-5′), 6.97 (1H, d, *J* = 8.4 Hz, H-6′), 3.77 (3H, s, 2′-OCH_3_), 3.74 (3H, s, 4′-OCH_3_); ^13^C-NMR (CD_3_OD), *δ*: 70.1 (C-2), 47.5 (C-3), 192.4 (C-4), 129.9 (C-5), 101.8 (C-6), 164.2 (C-7), 98.0 (C-8), 163.6 (C-9), 113.7 (C-10), 115.3 (C-1′), 157.7 (C-2′), 109.8 (C-3′), 160.0 (C-4′), 104.0 (C-5′), 128.5 (C-6′), 54.0 (2′-OCH_3_), 54.2 (4′-OCH_3_). These data were equal to those of literature [15].

*(3R)-Vestitone* (**2**). Yellow crystals; C_16_H_14_O_5_; ^1^H-NMR (CD_3_OD), *δ*: 4.40 (1H, dd, *J* = 11.0, 5.4 Hz, H-2a), 4.56 (1H, d, *J* = 11.0 Hz, H-2b), 4.12 (1H, dd, *J* = 11.0, 5.4 Hz, H-3), 7.74 (1H, d, *J* = 8.8 Hz, H-5), 6.48 (1H, dd, *J* = 8.8, 2.2 Hz, H-6), 6.31 (1H, d, *J* = 2.2 Hz, H-8), 6.38 (1H, d, *J* = 2.4 Hz, H-3′), 6.34 (1H, d, *J* = 8.4 Hz, H-5′), 6.88 (1H, d, *J* = 8.4 Hz, H-6′), 3.70 (3H, s, 4′-OCH_3_); ^13^C-NMR (CD_3_OD), *δ*: 72.0 (C-2), 48.7 (C-3), 194.7 (C-4), 130.4 (C-5), 111.7 (C-6), 166.4 (C-7), 103.6 (C-8), 165.8 (C-9), 115.7 (C-10), 115.8 (C-1′), 157.6 (C-2′), 102.7 (C-3′), 161.8 (C-4′), 106.0 (C-5′), 131.8 (C-6′), 55.7 (4′-OCH_3_). These data were identical to those reported [16,17,18].

*(3R)-2'*,*3'*,*7-Trihydroxy-4'-methoxyisoflavanone* (**3**). White powder; C_16_H_14_O_6_; ^1^H-NMR (CD_3_OD), *δ*: 4.49 (1H, d, *J* = 5.4 Hz, H-2a), 4.63 (1H, dd, *J* = 10.8, 5.4 Hz, H-2b), 4.17 (1H, dd, *J* = 10.8, 5.4 Hz, H-3), 7.78 (1H, d, *J* = 8.7 Hz, H-5), 6.53 (1H, dd, *J* = 8.7, 2.0 Hz, H-6), 6.35 (1H, d, *J* = 2.0 Hz, H-8), 6.45 (1H, d, *J* = 8.5 Hz, H-5′), 6.51 (1H, d, *J* = 8.5 Hz, H-6′), 3.83 (3H, s, 4′-OCH_3_); ^13^C-NMR (CD_3_OD, 100 MHz), *δ*: 71.9 (C-2), 48.7 (C-3), 194.5 (C-4), 130.4 (C-5), 111.7 (C-6), 149.1 (C-7), 104.2 (C-8), 165.6 (C-9), 116.8 (C-10), 115.5 (C-1′), 145.1 (C-2′), 135.2 (C-3′), 166.2 (C-4′), 103.6 (C-5′), 120.6 (C-6′), 56.6 (4′-OCH_3_). These data were consistent with those reported in [19].

*(3R)-4'-Methoxy-2'*,*3*,*7-trihydroxyisoflavanone* (**4**). White crystals; C_16_H_14_O_6_; ^1^H-NMR (acetone-*d*_6_), *δ*: 4.30 (1H, d, *J* = 11.8 Hz, H-2a), 4.88 (1H, d, *J* = 11.8 Hz, H-2b), 7.74 (1H, d, *J* = 8.7 Hz, H-5), 6.56 (1H, d, *J* = 8.7 Hz, H-6), 6.36 (3H, overlapped, H-3′, 5′, 8), 7.32 (1H, d, *J* = 9.3 Hz, H-6′), 3.67 (3H, s, 4′-OCH_3_); ^13^C-NMR (acetone-*d*_6_), *δ*: 75.5 (C-2), 76.0 (C-3), 191.6 (C-4), 131.6 (C-5), 112.8 (C-6), 166.5 (C-7), 104.4 (C-8), 164.9 (C-9), 114.5 (C-10), 118.9 (C-1′), 158.5 (C-2′), 104.1 (C-3′), 162.9 (C-4′), 106.7 (C-5′), 129.7 (C-6′), 56.4 (4′-OCH_3_). These data were in accordance with those reported in [9].

*Carthamidin* (**5**). White crystals; C_15_H_12_O_6_; ^1^H-NMR (CD_3_OD), *δ*: 5.44 (1H, dd, *J* = 13.0, 2.8 Hz, H-2), 2.83 (1H, dd, *J* = 17.1, 2.8 Hz, H-3a), 3.20 (1H, dd, *J* = 17.1, 13.0 Hz, H-3b), 6.05 (1H, s, H-8), 7.42 (2H, d, *J* = 8.5 Hz, H-2′, 6′), 6.96 (2H, d, *J* = 8.5 Hz, H-3′, 5′). These data were identical to those in the literature [20].

*Liquiritigenin* (**6**). White crystals; C_15_H_12_O_4_; ^1^H-NMR (CD_3_OD), *δ*: 5.58 (1H, dd, *J* = 13.2, 2.8 Hz, H-2), 2.95 (1H, dd, *J* = 16.9, 2.8 Hz, H-3a ), 3.26 (1H, dd, *J* = 16.9, 13.2 Hz, H-3b), 7.97 (1H, d, *J* = 8.7 Hz, H-5), 6.74 (1H, dd, *J* = 8.7, 2.2 Hz, H-6), 6.62 (1H, d, *J* = 2.2 Hz, H-8), 7.53 (2H, d, *J* = 8.6 Hz, H-2′, 6′), 7.08 (2H, d, *J* = 8.6 Hz, H-3′, 5′); ^13^C-NMR (CD_3_OD), *δ*: 80.3 (C-2), 44.5(C-3), 193.0 (C-4), 129.5 (C-5), 111.5 (C-6), 166.0 (C-7), 103.6 (C-8), 164.8 (C-9), 114.4 (C-10), 130.5 (C-1′), 128.4 (C-2′, 6′), 116.1 (C-3′, 5′), 158.1 (C-4′). These data were in accordance with those reported previously [19].

*Isoliquiritigenin* (**7**). Yellow crystals; C_15_H_12_O_4_; ^1^H-NMR (CD_3_OD), *δ*: 7.54 (3H, dd, *J* = 15.4, 6.0 Hz, H-2, 6, α), 6.88 (2H, d, *J* = 8.6 Hz, H-3, 5), 6.25 (1H, d, *J* = 2.4 Hz, H-3′), 6.37 (1H, dd, *J* = 8.8, 2.4 Hz, H-5′), 7.89 (1H, d, *J* = 8.8 Hz, H-6′), 7.73 (1H, d, *J* = 15.4 Hz, H-β); ^13^C-NMR (CD_3_OD) *δ*: 127.9 (C-1), 131.8 (C-2), 116.9 (C-3), 161.5 (C-4), 116.9 (C-5), 131.8 (C-6), 114.7 (C-1′), 166.4 (C-2′), 103.9 (C-3′), 167.5 (C-4′), 109.2 (C-5′), 133.4 (C-6′), 118.4 (C-α), 145.7 (C-β), 193.6 (C=O). These data were identical to those in the literature [15,19].

*(3R)-Vestitol* (**8**). White crystals; C_16_H_16_O_4_; ^1^H-NMR (CD_3_OD), *δ*: 3.93 (1H, t, *J* = 10.1 Hz, H-2a), 4.21 (1H, dd, *J* = 10.1, 4.1 Hz, H-2b), 3.42 (1H, m, H-3), 2.77 (1H, dd, *J* = 15.5, 4.1 Hz, H-4a), 2.93 (1H, dd, *J* = 15.5, 10.9 Hz, H-4b), 6.86 (1H, d, *J* = 8.2 Hz, H-5), 6.22 (1H, d, *J* = 2.4 Hz, H-8), 6.31 (1H, dd, *J* = 8.2, 2.4 Hz, H-3′), 6.37 (2H, m, H-6, 5′), 6.96 (1H, d, *J* = 8.2 Hz, H-6′), 3.71 (3H, s, 4′-OCH_3_); ^13^C-NMR (CD_3_OD), *δ*: 71.2 (C-2), 33.2 (C-3), 31.4 (C-4), 131.2 (C-5), 109.1 (C-6), 156.4 (C-7), 103.9 (C-8), 157.3 (C-9), 115.0 (C-10), 121.5 (C-1′), 157.5 (C-2′), 102.5 (C-3′), 160.9 (C-4′), 105.8 (C-5′), 128.8 (C-6′), 55.6 (4′-OCH_3_). These data were identical to those in the literature [19].

*Sulfuretin* (**9**). White crystals; C_15_H_10_O_5_; ^1^H-NMR (CD_3_OD), *δ*: 6.85 (1H, d, *J* = 8.2 Hz, H-4), 7.24 (1H, d, *J* = 8.2 Hz, H-5), 6.70 (3H, overlapped, H-7, 10, 6′), 7.54 (1H, s, H-2′), 7.61 (1H, d, *J* = 8.3 Hz, H-5′); ^13^C-NMR (CD_3_OD), *δ*: 147.7 (C-2), 184.4 (C-3), 126.9 (C-4), 116.8 (C-5), 169.9 (C-6), 99.4 (C-7), 168.7 (C-8), 114.8 (C-9), 114.8 (C-10), 125.4 (C-1′), 114.3 (C-2′), 146.8 (C-3′), 149.7 (C-4′), 119.0 (C-5′), 126.5 (C-6′). These data were consistent with those previously reported [21].

### 3.4. Bacterial Strains

The *R. solanacearum* strain was obtained from Professor Ming-he Mo of the Key Laboratory of Protection and Utilization of Biological Resources, Yunnan University, and maintained on a nutrient agar (NA) slant at 4 °C.

### 3.5. Antibacterial Activity

These compounds were individually tested for *in vitro* antibacterial activity against *R. solanacearum* strain by the filter paper disc agar diffusion method [22]. The NA medium was mixed with suspension (2 mL) containing 10^7^ CFU/mL of *R. solanacearum*, and then poured into Petri-plates to a uniform depth of 5 mm and was allowed to solidify. The isolated compounds dissolved in dimethyl sulfoxide (DMSO) (1.6 µL, 50 mg/mL) were impregnated on sterile filter paper discs (6 mm diameter) and then applied aseptically to the surface of the agar plates. Streptomycin sulfate (1.6 µL, 50 mg/mL) was used as positive control. The plates were incubated at 37 °C for 24 h. Then the diameters of the inhibition zones including the 6 mm disc diameter were measured. Experiments were done in triplicate, and the results were mean values.

## 4. Conclusions

In conclusion, a total of nine compounds including four isoflavanones **1**−**4**, two flavanones **5** and **6**, one chalcone **7**, one isoflavane **8** and one aurone **9** were isolated from *D. odorifera* and identified by comparison of their NMR data with data reported in the literature. In addition, all compounds were evaluated for their inhibitory activity against *R. solanacearum*. Among the nine flavonoids, compound **8** exhibited the strongest antibacterial activity and compounds **2**, **6**, and **7** showed strong antibacterial activity. This is the first report of the anti-*R. solanacearum* activity of the compounds from *D.odorifera*.
